# Factors that improve the adherence to medication in asthmatic patients treated with inhalation therapy

**DOI:** 10.1186/1939-4551-8-S1-A254

**Published:** 2015-04-08

**Authors:** Vincenzo Patella, Giovanni Florio

**Affiliations:** 1 Site of School Network in Allergy and Clinical Immunology, Italy

## Background

Adherence to inhalation therapy is a critical determinant of the success of asthma management. However, in practice, no adherence to inhalation therapy is very common in asthmatics. The effects of adherence to inhalation therapy in asthma is less known about the relationship between medication adherence and quality of life in these patients. The aim of this study is to assess the factors that contribute to adherence to inhalation therapy and examine their correlation with quality of life.

## Methods

In 264 asthmatics, enrolled following GINA guideline, a cross-sectional analysis was performed using a self-reported adherence questionnaire with responses on a 7-point Likert scale (7 = not impaired at all - 1 = severely impaired).

## Results

Of the 264 patients who were potential participants, 175 (66.3%) responded with usable information. The only significant factor associated with the overall mean adherence score was receiving repeated instruction about inhalation techniques (P = 0.037). Of the 175 respondents, 84 (48.0%) were given repeated verbal instruction and/or demonstrations of inhalation technique by a physician. Significant correlations were found between the overall mean adherence score and the health-related quality of life score (Asthma Quality of Life Questionnaire (AQLQ): total, r = −0.37, P = 0.028; symptoms, r = −0.41, P = 0.001; impacts, r = −0.38, P = 0.002). Furthermore, patients with repeated instruction showed better quality of life scores than those who did not receive instruction (total, P = 0.033; symptoms, P = 0.031; impacts, P = 0.021).

## Conclusions

Repeated instruction for inhalation techniques may contribute to adherence to therapeutic regimens, which relates to better health status in patients suffering of asthma.

